# Cancer Risk After Initial Surveillance of Intraductal Papillary Mucinous Neoplasms (IPMNs): Are We Missing the Window for Prevention?

**DOI:** 10.1245/s10434-026-19898-2

**Published:** 2026-06-09

**Authors:** Antonio Pea, Tommaso Dall’Olio, Dario Solinas, Chiara Filippini, Elisa Venturini, Matteo De Pastena, Claudio Luchini, Stefano Crinò, Giulia A. Zamboni, Mirko D’Onofrio, Roberto Salvia

**Affiliations:** 1 General and Pancreatic Surgery Unit, Pancreas Institute, University of Verona, Verona, Italy; 2 Department of Diagnostics and Public Health, Section of Pathology, University of Verona, Verona, Italy; 3 Gastroenterology and Digestive Endoscopy Unit, Pancreas Institute, University of Verona, Verona, Italy; 4 Radiology Unit, Pancreas Institute, University of Verona, Verona, Italy

## Abstract

**Background:**

The management of intraductal papillary mucinous neoplasms (IPMNs) has evolved through successive International Association of Pancreatology guidelines, aiming to refine surgical indications and improve cancer prevention. With broader adoption of surveillance, the oncologic outcomes of patients resected after follow-up—and the effectiveness of current strategies in preventing malignancy—remain unclear. This study examined how evolving management has influenced surgical selection and cancer prevention, particularly by comparing patients resected at diagnosis versus after surveillance.

**Methods:**

Patients with presumed IPMN across four International Association of Pancreatology guideline periods (pre-2006, 2006–2012 [Sendai], 2012–2017 [Fukuoka], and 2017–2024 [Fukuoka revisions]) were analysed by clinical trajectory: follow-up without surgery, upfront surgery (resection within 12 months of diagnosis), and post-surveillance resection (PR). Endpoints included surgical indications and rates of low-grade dysplasia (LGD), high-grade dysplasia (HGD), and invasive carcinoma (IC).

**Results:**

Across guideline periods, patients managed with surveillance increased from 172 before 2006 to 828 in 2006–2012, 1,572 in 2012–2017, and 1,193 after 2017, while PR increased from 11 to 29, 60, and 204, respectively. Overall, among 3,304 patients, 2,452 (74%) were managed with surveillance, 548 (17%) underwent upfront surgery, and 304 (9%) had PR. In the PR group, resections for a single high-risk stigmata (HRS) increased from 9% to 48%, and those with multiple HRS up to 20% after 2017. At pathology, LGD decreased from 46% to 17%, whereas HGD and IC increased from 18 to 28% and from 36 to 45%, respectively. In the upfront surgery group, LGD decreased from 49% to 26%. Development of HRS during follow-up was associated with a higher risk of HGD/IC (odds ratio 2.18, *p* = 0.008).

**Conclusions:**

While evolving IPMN management has reduced rates of LGD and increased detection of HGD, invasive carcinoma remains frequent at resection after surveillance, as surgery is often delayed until HRS emerge. Improved tools are needed to optimize timing and define appropriate oncologic outcomes.

**Supplementary Information:**

The online version contains supplementary material available at 10.1245/s10434-026-19898-2.

Intraductal papillary mucinous neoplasms (IPMNs) are precursor lesions of pancreatic cancer. While early detection and timely resection can improve outcomes, most IPMNs do not progress to malignancy, and the majority of patients will not require surgery during their lifetime.^1,2^ Over the years, international consensus guidelines, primarily those issued by the International Association of Pancreatology (IAP) in 2006, 2012, 2017, and more recently in 2024, have aimed to identify patients at risk for malignant transformation. These guidelines rely on clinical criteria, such as worrisome features (WF) and high-risk stigmata (HRS),^3–6^ with the goal of identifying lesions harbouring or at risk of evolving into cancer, while avoiding unnecessary resections.

They have been predominantly based on surgical series and static assessments of risk features, with high-grade dysplasia (HGD) and invasive carcinoma (IC) as the main endpoints for detection.^7^

As surveillance has become more widely adopted, international guidelines have gradually refined surgical criteria to balance cancer prevention with the risk of overtreatment. Yet, limited data are available on how these criteria have shaped real-world management and oncological outcomes—particularly in patients initially selected for surveillance.

This study therefore analysed IPMN management at the Pancreas Institute of Verona across successive IAP guideline periods, evaluating whether evolving clinical practice improved surgical selection and cancer prevention. We compared oncologic outcomes in patients resected at diagnosis and those resected after surveillance, under the assumption that evolving risk-features may reflect biological progression. By analysing trends in clinical decision-making and pathology over time, we aimed to identify limitations in current risk stratification and inform future improvements.

## Methods

### Patients’ Selection

A prospectively maintained database of patients with pancreatic cysts evaluated at the Pancreas Institute of Verona between 1993 and 2024 was queried for cases of presumed IPMNs, managed either surveillance or surgical intervention.

Clinical management during each period followed the International Association of Pancreatology consensus guidelines available at the time: Sendai (2006)^3^; Fukuoka (2012)^4^; their revision (2017)^5^; and the latest 2024 guidelines^6^ (Supplementary Fig. S1).

For each patient, the most recent clinical assessment within the corresponding guideline period was considered; in resected patients, the final preoperative visit was used. As a result, each patient could contribute one visit per period to the time-trend analysis. This approach was intended to reflect the decision-relevant clinical status within each era (continued surveillance vs. preoperative assessment) rather than to model within-patient disease progression over time.

Data collection included demographic, clinical, radiological, and histopathological information, presumptive diagnosis and management decision (surgery or continued surveillance). Radiological evaluation was based on imaging modalities, such as computed tomography (CT) scan, magnetic resonance imaging (MRI), or endoscopic ultrasonography (EUS).

Features assessed included cyst location, size (with thresholds: >3 and >4 cm), multifocality, main pancreatic duct (MPD) caliber (5–9 mm or >10 mm), septations, mural nodules, solid components, wall thickening, Ca 19.9, and lymphadenopathy. For multifocal cysts, the largest or most concerning lesion was considered.

Histopathology in resected cases was classified as low-grade dysplasia (LGD), HGD, or IC.^8,9^ Cases with final diagnoses other than IPMN were classified as “other.”^10^ The study complied with Regulation (EU) 2016/679 on data protection and Italian law (Gazzetta Ufficiale No. 72, March 26, 2012) on anonymized health data. Formal consent was waived due to the retrospective and anonymized design. The surgical database was approved by the Verona Ethics Committee (protocol PAD-R, n.1101CESC).

### Time Trend Analysis

Patients with presumptive IPMNs were stratified into three groups based on management:

1. Follow-up (FU): Patients managed exclusively with radiological surveillance.
2. Upfront surgery (UF): Patients who underwent surgery within 12 months of initial clinical diagnosis. As in previous studies, this 12-month threshold was used to distinguish immediate surgery from true surveillance.^11^ This timeframe accounts for the period typically needed for baseline evaluation, including referral from lower-volume centres and second-level imaging. It also reflects surgical waiting times in high-volume centres, making it a reliable marker to differentiate upfront surgical management from surveillance-based decisions.
3. Post-surveillance resection (PR): Patients initially under surveillance who underwent surgery owing to clinical or radiological progression.

Clinical risk features at each visit were categorized into four groups:

- "None" for the absence of WFs;
- "Cumulative WFs" for multiple WFs;
- "1 HRS ± WF" for a single HRS regardless of additional WFs;
- "Cumulative HRS” for more than one HRS.

### Upfront Surgery vs. Post-Surveillance Resections

Patients in the UF group were compared with those in the PR group. Risk features from the final preoperative visit were analysed to determine whether features present at diagnosis (UF) versus those developing during follow-up (PR) differed in their predictive value for high-risk lesions (HGD and IC) or for IC alone.

### Statistical Analysis

Categorical variables were reported as frequencies and percentages; continuous variables as medians with interquartile ranges (IQR). Group comparisons used the chi-square or Fisher’s exact test for categorical data and the Kruskal-Wallis test for continuous variables.

Univariate logistic regression assessed associations between clinical features and pathology, using UF patients as the reference group. Odds ratios (OR) with 95% confidence intervals (CI) were calculated for WFs and HRS present at diagnosis or emerging during follow-up. Statistical significance was defined as a two-tailed *p* < .05. Analyses were performed using SPSS version 29.

## Results

### Study Population Characteristics

**Fig. 1:**
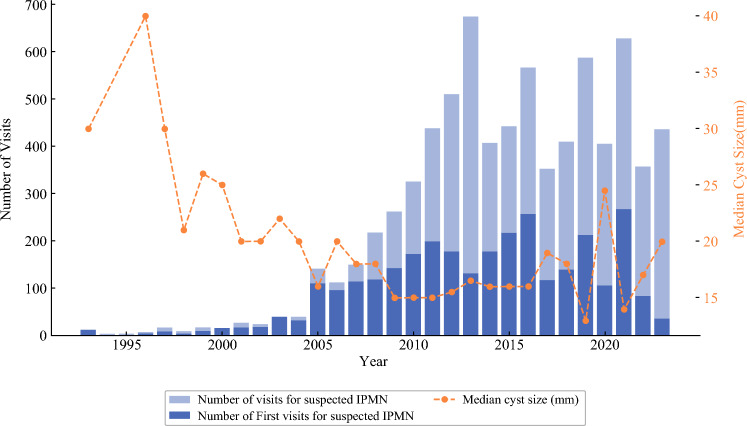
Trends in follow-up (FU) and first visits for suspected IPMN, showing an increase in FU visits and a decrease in median cyst size over time during the study periods

Between 1993 and 2024, 3,304 patients underwent clinical evaluation for suspected IPMN (Tables 1 and S1). Of these, 2,452 (74.2%) were managed through surveillance, 304 (9.2%) underwent surgery during surveillance (PR group), and 548 (16.6%) had upfront surgery after initial workup (UF group). A total of 8,229 follow-up visits were recorded. For temporal trend analysis, 4,617 visits were included by selecting one visit per patient per guideline period (Table S2). Follow-up visits increased over time, with a median annual growth rate of 1.84% (Fig. 1).

**Table 1:** Study population and comparison between cohorts population characteristics pre-guidelines, 2006–2012, 2012–2017, after 2017. Patients were classified into three groups: under surveillance (FU, follow-up without surgery), undergoing upfront surgery (UF, resection within 12 months from diagnosis), or undergoing surgery after an initial period of surveillance (PR, post-surveillance resection performed > 12 months after diagnosis). The first column reports overall patient characteristics, while subsequent columns reflect data based on one clinical visit per patient per time period

	Overall patients	< 2006	2006-2012	2012-2017	2017-2024	*p* value (across guideline periods)
Gender, male (%)	1,526 (46.2)	144 (45.4)	392 (39.8)	656 (37.1)	684 (44.2)	< 0.001
Age, median (IQR)	67 (59–73)	64 (55–70)	67 (59–73)	68 (60–74)	68 (60–-75)	< 0.001
Cyst location (%)						< 0.001
Head	1,742 (52.7)	192 (60.6)	490 (49.7)	798 (45.2)	628 (40.6)	
Body	946 (28.6)	59 (18.6)	261 (26.5)	475 (26.9)	459 (29.7)	
Tail	273 (8.3)	46 (14.5)	144 (14.6)	306 (17.3)	296 (19.1)	
Entire pancreatic gland	344 (10.4)	20 (6.3)	90 (9.1)	187 (10.6)	165 (10.7)	
Jaundice, yes (%)	92 (2.8)	6 (1.9)	20 (2.0)	34 (1.9)	36 (2.3)	NS
Acute pancreatitis, yes (%)	235 (7.1)	26 (8.2)	57 (5.8)	108 (6.1)	108 (7.0)	NS
Ca 199 elevated	125 (3.8)	17 (5.4)	41 (4.2)	65 (3.7)	48 (3.1)	NS
(Normal <37 UI/mL) (%)						
Cyst type (suspected) (%)						<0.001
BD-IPMN	2,480 (75)	222 (70.0)	839 (85.2)	1,510 (85.5)	1,139 (73.6)	
MD-IPMN	198 (6)	39 (12.3)	38 (3.9)	49 (2.8)	84 (5.4)	
MT-IPMN	626 (19)	56 (17.7)	108 (11.0)	207 (11.7)	325 (21.0)	
Cyst size, > 30 mm (%)	772 (23.4)	91 (28.7)	211 (21.4)	352 (19.9)	339 (21.9)	0.009
Cyst size, median (IQR)		20 (12–30)	18 (10–26)	16.5 (10–25)	18 (10–27)	<0.001
Main duct dilatation (>5mm), yes (%)	809 (24.5)	58 (18.3)	91 (9.2)	177 (10.0)	266 (17.2)	<0.001
Main duct dilatation (>10mm), yes (%)	227 (6.9)	38 (12.0)	89 (9.0)	109 (6.2)	89 (5.7)	<0.001
Mural nodule /Solid component, yes (%)	272 (8.2)	24 (7.6)	53 (5.4)	109 (6.2)	142 (9.2)	<0.001
EUS performed, yes (%)	692 (20.9)	7 (2.2)	39 (4.0)	205 (11.6)	352 (22.7)	<0.001
Management (%)						<0.001
Surveillance	2,452 (74.2)	172 (54.3)	828 (84.1)	1,572 (89.0)	1,193 (77.0)	
Surgery UF	548 (16.6)	134 (42.3)	128 (13.0)	134 (7.6)	152 (9.8)	
Surgery PR	304 (9.2)	11 (3.5)	29 (2.9)	60 (3.4)	204 (13.2)	
Total	3,304^a^	317^b^	985^b^	1,766^b^	1,549^b^	

^a^Total of patients enrolled considered in the study

^b^Total amount of clinical visits within time period considering the limit of one visit per patient per time period

BD-IPMN: branch duct IPMN, MD-IPMN: main duct IPMN, MT-IPMN mixed-type IPMN, EUS: endoscopic ultrasound, UF: upfront, PR post-surveillance

Most patients were diagnosed with branch-duct IPMN (BD-IPMN, 2,480; 75%), followed by mixed-type (MT-IPMN, 626; 19%) and main-duct IPMN (MD-IPMN, 198; 6%). Clinically, 92 patients (2.8%) presented with jaundice, 235 (7.1%) experienced acute pancreatitis, and 125 (3.8%) had elevated Ca1-.9 levels (>37 UI/mL).

The most common high-risk features were cyst size >30 mm (772; 23.4%), main duct dilation of 5–9 mm (582; 17.6%), or >10 mm (227; 6.9%). Mural nodules or solid components were identified in 272 patients (8.2%).

### Patient Characteristics Across Guideline Periods

Patient demographics, radiological findings, and management strategies varied significantly across guideline periods (Table 1). The number of patients evaluated increased from 317 pre-2006 to 1,549 in 2017–2024. Median age at presentation rose from 64 years (IQR 55–70) to 68 years (IQR 60–75) (*p* < 0.001).

Cysts were predominantly located in the pancreatic head in all periods. BD-IPMNs remained the most frequent subtype, increasing to 73.6% after 2017, while MT-IPMNs rose from 17.7% to 21% (*p* < 0.001). Median cyst size decreased from 20 mm (IQR 12–30) to 18 mm (IQR 10–27) (*p* < 0.001), and the rate of cysts >30 mm declined from 28.7% to 21.9% (*p* = 0.009).

Main duct dilatation (>5 mm) was seen in 18.3% of patients pre-2006 and 17.2% after 2017 (*p* < 0.001). Mural nodules or solid components were reported in 7.6% pre-2006 and 9.2% after 2017 (*p* < 0.001).

Use of EUS increased markedly from 2.2% to 22.7% (*p* < 0.001) (Fig. S3). Surveillance became the predominant approach, rising from 54.3% to 77.0% (*p* < 0.001). The proportion of patients undergoing surgery after surveillance increased from 3.5% to 13.2% (*p* < 0.001).

### Clinical Risk Features Across Guideline Periods

**Fig. 2:**
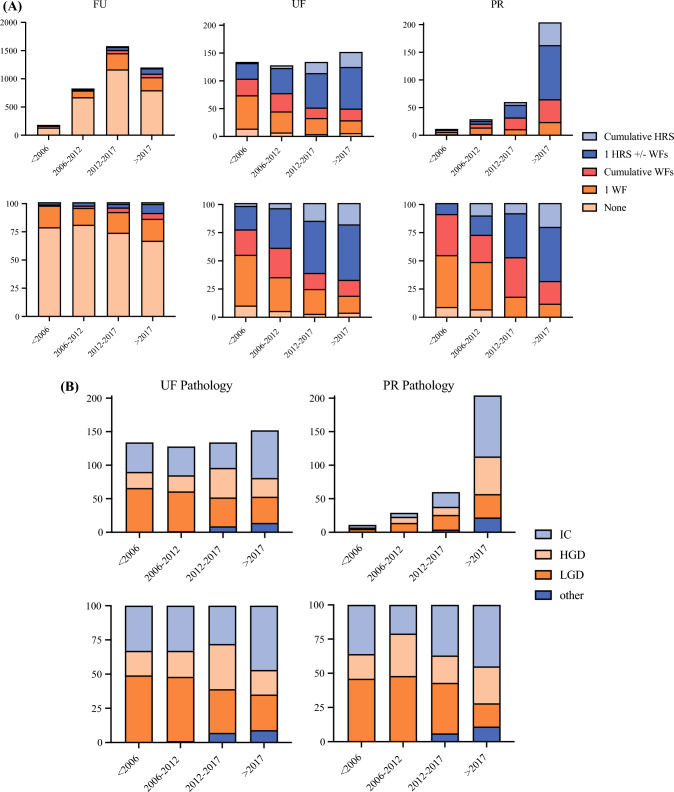
**A** Distribution of clinical risk features across three patient cohorts: those managed with surveillance (FU, follow-up), those undergoing upfront surgical resection (UF), and those undergoing surgery after more than 12 months of surveillance due to progression (PR, post-surveillance resection). Risk features are categorized as worrisome features (WF) and high-risk stigmata (HRS). **B** Distribution of pathological outcomes for IPMN patients in the UF and PR groups across different time periods. Pathological diagnoses include low-grade dysplasia (LGD), high-grade dysplasia (HGD), invasive carcinoma (IC), and other non-IPMN findings. Data for panels A and B are detailed in Tables S2 and S4 of supplementary material, respectively

The distribution of clinical risk features changed significantly across guideline periods (Fig. 2A; Tables S3 and S4). Among 2,452 patients under surveillance (FU group), 1,600 were actively followed at our institution (median follow-up: 43 months; IQR 20–80). Although the absolute number of patients without risk features increased from 135 pre-2006 to 797 after 2017, their proportion declined from 78% to 67%. Patients with one WF rose from 33 to 232, remaining stable at 19%. Cumulative WFs increased from two (1%) to 62 (5%), while those with 1 HRS ± WF increased from two (1%) to 96 (8%). Cumulative HRS, not seen before 2017, were observed in five patients (0.4%).

Among UF patients, resections in those without risk features decreased from 14 (10%) pre-2006 to six (4%) after 2017. Surgeries for patients with one WF and cumulative WFs declined from 60 (45%) to 23 (15%) and from 30 (22%) to 21 (14%), respectively. In contrast, surgeries in patients with one HRS ± WF increased from 28 (21%) to 75 (49%).

In the PR group (median follow-up: 54 months; IQR 29–84), patients with 1 WF increased from five to 24 (12%), and those with cumulative WFs to 41 (20%). Patients with one HRS ± WF increased from one (9%) pre-2006 to 98 (48%) after 2017; cumulative HRS, previously absent, were observed in 41 patients (20%).

An increasing number of MD- and MT-IPMN cases remained under surveillance over time, with a growing proportion eventually undergoing surgery (Supplementary Fig. S2).

### Changes in Oncological Outcomes in Surgical Patients

In the UF group, LGD at final pathology decreased from 66 (49%) pre-2006 to 29 (26%) after 2017, while IC increased from 44 (33%) to 71 (47%). The proportion of HGD remained relatively stable (~18–19%).

In the PR group, the proportion of LGD declined from 45% (5 cases) to 17% (35 cases). IC cases increased from 4 (36%) to 91 (45%), and HGD from 2 (18%) to 56 (28%) after 2017 (Fig. 2B; Table S5).

### Impact of Emerging Risk Features on Oncological Outcomes

We assessed whether risk features developing during surveillance had different prognostic value than those present at diagnosis. Patients who developed one WF had a higher, though nonsignificant, risk of HGD/IC compared with UF patients with one WF (OR 1.831; *p* = 0.077). In contrast, developing one HRS ± WF was significantly associated with increased HGD/IC risk compared to HRS at diagnosis (OR 2.18; *p* = 0.008). The timing of surgery did not significantly influence HGD/IC risk in patients with cumulative WFs or cumulative HRS. When risk features were analysed for IC alone vs. LGD/HGD, no variables were significantly associated with IC development (Table 2).

**Table 2:** Effect size of clinical risk features on the development of high-grade dysplasia (HGD) / invasive carcinoma (IC) (A) and invasive carcinoma only (B) in patients undergoing upfront (UF) surgery vs. post-surveillance resection (PR)

Clinical Risk Features	LGD vs HGD/IC		LGD/HGD vs IC	
	OR (95%CI)	P value	OR (95%CI)	P value
1 WF				
UF	Ref		Ref	
PR (>12 months)	1.831 (0.936-3.585)	0.077	1.766 (0.900-3.467)	0.098
Cumulative WFs				
UF	Ref		Ref	
PR (>12 months)	1.110 (0.593-2.079)	0.744	1.107 (0.584-2.097)	0.756
1 HRS +/- WFs				
UF	Ref		Ref	
PR (>12 months)	2.179 (1.222-3.886)	0.008	1.283 (0.806-2.043)	0.294
Cumulative HRS				
UF	Ref		Ref	
PR (>12 months)	0.404 (0.127-1.284)	0.124	0.529 (0.238-1.173)	0.117

WF: Worrisome feature, HRS: high risk stigmata, UF: surgery upfront, PR: surgery post-surveillance, LGD: low-grade dysplasia, HGD: high-grade dysplasia, IC: invasive carcinoma

## Discussion

This study analyses the evolution of clinical management of IPMNs at the Pancreas Institute of Verona, highlighting the impact of successive IAP guidelines on patient selection for surveillance and surgery and the associated oncological outcomes.

Over nearly two decades, the number of patients evaluated for suspected IPMN in Verona increased, driven by advancements in cross-sectional imaging^12,13^ and a better understanding of the disease.^14,15^ This led to an increased detection of incidental pancreatic cysts, a progressive annual rise in the number of follow-up visits, and a corresponding increase in the number of patients undergoing surgery after a period of surveillance.

Management of IPMN, as guided by successive IAP recommendations, has aimed to improve risk stratification, with surgical indications focused on targeting high-risk lesions (namely HGD and IC) at final pathology.^16^ Pulvirenti et al. previously assessed the impact of guideline implementation on surgical selection for IPMNs in tertiary centres. Despite an increasing prevalence of WFs and HRSs in surgical cohorts, the HGD/IC rate in BD-IPMN remained low (<30%) and did not vary significantly between institutions, likely reflecting consistent surgical indications despite variability in radiological interpretation.^17^ Our study focused on radiological characteristics rather than IPMN subtypes, thereby reducing classification variability and minimizing reporting discrepancies across time periods.

While this and other studies^16–18^ that have evaluated the evolution of IPMN guidelines have reported progressively improved specificity and positive predictive value for detecting HGD/IC at final pathology, they are largely limited to surgical cohorts with histological confirmation. Therefore, they do not account for the clinical context that prompted resection and fail to distinguish between patients resected at diagnosis and those undergoing surgery after a period of surveillance.

Key aspects of this study are the inclusion of patients managed with surveillance, reflecting current clinical practice, along with the separate analysis of oncological outcomes in patients undergoing upfront surgery (UF group)—typically with clinical risk-features already present at diagnosis—versus those resected after a period of surveillance (PR group), in whom risk features developed over time.

The outcomes of these two groups require distinct interpretation: IC in the UF group may reflect appropriate surgical indication, while IC in the PR group—following a period of surveillance aimed at intercepting malignant transformation—may reflect a later stage of intervention. Without accounting for these clinical trajectories, evaluations of guidelines performance may lead to overtreatment or underestimate missed cancers, depending on the context. However, the observed patterns likely reflect both evolving surgical decision thresholds over time and the natural history of IPMN progression, rather than a direct effect of management strategy alone.

In our surveillance cohort, we observed an increase in patients monitored with WFs (one or cumulative, 24% after 2017) and those with one HRS (8%). In the UF group, surgeries for WFs declined, reflecting a shift toward initial surveillance for these patients. This likely stems from uncertainty about whether these features represent neoplastic progression or stable morphology. The HRS remained consistent across time periods, continuing to serve as absolute indicators for surgery.

Patients transitioning from surveillance to surgery (PR group) increased in recent years, also reflecting this shift in practice. This cohort showed a growing prevalence of WFs and HRS (up to 68% after 2017). While this study did not assess progression patterns from diagnosis, these findings may reflect limitations in earlier literature and guidelines in determining the optimal timing for surgery in evolving IPMNs,^19^ as well as a growing tendency to delay intervention until high-risk stigmata emerge. In this setting, EUS has gained increasing relevance and was performed in 23.3% of all IPMN cases at our institution after 2017.^20,21^ Although the 2024 IAP guidelines do not mandate its use for surgical decision-making,^6^ in our practice, EUS is selectively employed to assess suspected HRS—particularly mural nodules—and to confirm or delay surgical indications at the time of evaluation. This targeted use may have influenced the timing of surgery in patients initially managed with surveillance.

Pathological outcomes showed a reduction in LGD resections in the UF group, indicating improved patient selection and fewer unnecessary surgeries, likely also influenced by reduced surgical indications for WFs. Conversely, the PR group had a significant increase in HGD, representing the ideal target, and IC after 2017, suggesting that surveillance strategies may not always prompt surgery at an earlier stage of disease progression.

Because the onset of WFs present at diagnosis cannot be determined—and 23% of patients in our cohort were managed with surveillance—this raises the question of whether the predictive value of clinical risk-features for cancer differs when they emerge during follow-up rather than being present at baseline.

Comparing UF and PR groups, only the emergence of HRS during surveillance was significantly associated with HGD/IC at final pathology, in line with prior studies showing that progressing features—main duct dilation, cyst growth, and mural nodules—are associated with increased malignancy.^11,22,23^ In contrast, WFs, whether present at diagnosis or developing later, were not clearly associated with higher yield, suggesting that WFs—despite indicating progression—may still lead to surgery for LGD. Conversely, multiple HRS, whether present at diagnosis or emerging during follow-up, were associated with similarly high cancer rates.

Our data underscore the difficulty in distinguishing HGD from IC, as no clinical features were uniquely associated with cancer—potentially limiting the window for intervention to target HGD.^9^ This supports the need for dynamic risk models and highlights the challenge of determining the optimal surgical timing in patients under surveillance—balancing early intervention for LGD against delaying surgery until HRS emerge, when the likelihood of detecting high-risk lesions, including cancer, is higher.

In this context, the definition of appropriate oncological outcomes must align with the patient’s clinical trajectory. For those undergoing upfront surgery, HGD or IC represents the intended surgical target. Conversely, for patients managed initially with surveillance, a resection yielding LGD may reflect timely intervention and successful prevention of malignant transformation.

These findings underscore the need for reliable biological markers^24–26^ to support surgical decision-making and highlight the substantial radiological overlap between HGD and IC, which complicates risk stratification. Prospective models integrating lesion kinetics, molecular profiling, and AI-based imaging may enhance early identification of malignant progression and optimise surgical timing. Molecular analysis of cyst fluid has emerged as a promising adjunct in the management of pancreatic cystic lesions.^27,28^ Recent molecular panels have demonstrated high diagnostic accuracy in classifying pancreatic cysts; however, their ability to predict early biological progression and guide the timing of intervention during surveillance remains not completely defined. In our healthcare system, routine implementation is currently limited by heterogeneous access to validated assays and the absence of reimbursement within publicly funded frameworks. Our findings support the development of dynamic models integrating imaging and molecular tools to better define the window for cancer prevention.

This study has several limitations, primarily owing to its retrospective nature, which may have led to missing data, particularly in earlier time periods. Advancements in imaging and evolving interpretation of radiological features over time may have influenced risk classification. While preoperative imaging was consistently available in recent years, earlier cases often relied on clinical or radiological reports. Similarly, shifts in pathological terminology may have affected histological classification, potentially underestimating cancers arising from IPMNs. Patterns of clinical progression during surveillance and the role of EUS in confirming or delaying surgery were not analysed in detail. Adherence to surveillance protocols and follow-up intensity were not systematically evaluated, possibly influencing the timing of risk-feature detection.

Another limitation relates to differences in surveillance duration across patients and guideline periods. Because time-trend analyses were based on one visit per patient per period (the most recent), later eras may include patients with longer follow-up, allowing greater opportunity for risk features or invasive carcinoma to emerge. Consequently, increases in high-risk features and malignancy over time may partially reflect differences in time-at-risk in addition to evolving management strategies.

Additionally, this study does not account for minimally-invasive cancers, which are associated with better prognosis following resection,^29^ while concomitant PDAC and recurrences in the pancreatic remnant were excluded, possibly underestimating the overall oncologic burden of IPMNs.^30–32^

## Conclusions

This study highlights the evolving management of IPMNs under successive IAP guidelines, demonstrating improved surgical selection and increased reliance on surveillance. However, challenges persist in determining the optimal timing for surgery, as a substantial rate of IC at final pathology remains, particularly among patients initially selected for surveillance. While HRSs remain key surgical indicators, WFs alone—even when emerging during follow-up—have limited predictive value for high-risk lesions. These findings emphasise the need for improved risk stratification tools, including refined dynamic criteria to better predict malignant progression and optimise surveillance strategies.

## Supplementary Information

Below is the link to the electronic supplementary material.

Supplementary file1 (DOCX 452 KB)
