# Supplementary material for: Cancer Risk After Initial Surveillance of Intraductal Papillary Mucinous Neoplasms (IPMNs): Are We Missing the Window for Prevention?
Source: Ann Surg Oncol. 2026 Jun 9;33(9):8429–37. doi: 10.1245/s10434-026-19898-2 (PMC13452840; doi:10.1245/s10434-026-19898-2)
Supplement: Supplementary file 1 [file 10434_2026_19898_MOESM1_ESM.docx]

**Supplementary materials**

**Figure S1: Summary of IPMN Guidelines (2006–2024). Comparison of key recommendations from the 2006, 2012, 2017, and 2024 IAP guidelines, including definitions of worrisome features (WFs) and high-risk stigmata (HRSs) and surgical indications.**

**Table S1: Study Population Characteristics**

|  | **Overall population (n=3304)** |
| --- | --- |
| Gender, male | 1526 (46.2) |
| Age, median (IQR) | 67 (59-73) |
| Cyst location  Head  Body  Tail  Entire pancreas | 1742 (52.7)  946 (28.6)  273 (8.3)  344 (10.4) |
| Jaundice | 92 (2.8) |
| Acute pancreatitis | 235 (7.1) |
| Ca 19.9 elevation  (Normal <37 UI/mL) | 125 (3.8) |
| Mural nodule /Solid component | 272(8.2) |
| Main pancreatic duct dilatation (>5mm) | 809 (24.5) |
| Cyst type (suspected)     BD-IPMN  MD-IPMN  MT-IPMN | 2480 (75)  198 (6)  626 (19) |
| Cyst size, >30 mm | 772 (23.4) |
| Management  Surveillance  Surgery | 2452 (74.2)  852 (25.8) |
| EUS, performed | 692 (20.9) |
| Abbreviations: branch duct IPMN (BD-IPMN); main duct IPMN (MD-IPMN); mixed-type IPMN (MT-IPMN); endoscopic ultrasound (EUS) | |

**Table S2: Total and analysed clinical visits across patient groups.**

Clinical visits recorded during surveillance for each group: patients managed with surveillance (FU group), those undergoing upfront surgery after diagnosis (UF group), and those undergoing surgery after a period of surveillance (PR group). A total of 8,229 follow-up visits were recorded: 6,904 in the surveillance group, 617 in the UF group, and 708 in the PR group.

For the purposes of longitudinal analysis, we included only in-person clinical visits conducted at the dedicated pancreatic cyst clinic of our institution. We excluded visits documented solely through phone, email, or informal communications, as well as diagnostic-only encounters such as isolated EUS, imaging studies, or laboratory tests not accompanied by a formal clinic assessment. To ensure consistency, we limited the analysis to one visit per patient per time period, selecting the most recent visit within each interval. In patients who underwent surgery, the final preoperative visit served as the reference. This approach yielded a total of 4,617 analyzed visits.

A total of 1,600 patients received active longitudinal surveillance at our center, and survival data were available for nearly all patients through regional health records.

Among the 2,452 patients initially enrolled in surveillance, 1,600 received active longitudinal follow-up at our institution (median follow-up 43 months, IQR 20–80). Of these, 155 were followed for more than 10 years, and other 31 underwent surgery after over a decade of surveillance.

| **Group** | **Total visits (All)** | **Analysed visits (1 per time period)** |
| --- | --- | --- |
| **Surveillance (FU)** | 6,904 | 3,724 |
| **Upfront Surgery (UF)** | 617 | 550 |
| **Post-Surveillance Surgery (PR)** | 708 | 343 |
| **Total** | **8,229** | **4,617** |

Among the remaining patients, 721 were referred for a single consultation (e.g., for second opinion or diagnostic confirmation) and continued follow-up elsewhere, while others exited surveillance over time due to comorbidities, advanced age, or patient preference.

**Table S3: Distribution of clinical risk features across the three management cohorts: patients under surveillance (FU), patients undergoing ex-novo resection (EN), and patients undergoing post-surveillance resection (PR). This table corresponds to the data illustrated in Figure 2A.**

|  | **<2006** | | | **2006-2012** | | | **2012-2017** | | | **>2017** | | |
| --- | --- | --- | --- | --- | --- | --- | --- | --- | --- | --- | --- | --- |
|  | **FU** | **EN** | **PR** | **FU** | **EN** | **PR** | **FU** | **EN** | **PR** | **FU** | **EN** | **PR** |
| **None** | 135 (78%) | 14 (10%) | 1  (9%) | 672 (81%) | 7  (5%) | 2  (7%) | 1165 (74%) | 4  (3%) | 0  (0%) | 797 (67%) | 6  (4%) | 0  (0%) |
| **1 WF** | 33 (19%) | 60 (45%) | 5  (45%) | 121 (15%) | 38 (30%) | 12 (41%) | 289 (18%) | 29 (22%) | 11 (18%) | 232 (19%) | 23 (15%) | 24  (12%) |
| **Cumulative WFs** | 2  (1%) | 30 (22%) | 4  (36%) | 14  (2%) | 33 (26%) | 7  (24%) | 58  (4%) | 19 (14%) | 21 (35%) | 62  (5%) | 21 (14%) | 41  (20%) |
| **1 HRS +/- WFs** | 2  (1%) | 28 (21%) | 1  (9%) | 21  (3%) | 45 (35%) | 5  (17%) | 54  (3%) | 62 (46%) | 23 (38%) | 96  (8%) | 75 (49%) | 98  (48%) |
| **Cumulative HRS** | 0  (0%) | 2  (1%) | 0  (0%) | 0  (0%) | 5  (4%) | 3  (10%) | 6  (0%) | 20 (15%) | 5  (8%) | 5  (0%) | 27 (18%) | 41  (20%) |
| **Total** | 172 | 134 | 11 | 828 | 128 | 29 | 1572 | 134 | 60 | 1193 | 152 | 204 |
| Abbreviations: worrisome feature (WF); high risk stigmata (HRS); follow-up group (FU); surgery ex-novo (EN); surgery post-surveillance (PR) | | | | | | | | | | | | |

**Table S4: Distribution of single WF or HRS in resected patients across different time periods. Each patient could have more than one WF or HRS**

| **Variable** | **<2006** | **2006-2012** | **2012-2017** | **2017-2023** |
| --- | --- | --- | --- | --- |
| **Jaundice** | 4 (3%) | 9 (6%) | 23 (12%) | 42 (12%) |
| **MN/Solid component** | 11 (8%) | 21 (13%) | 54 (28%) | 140 (39%) |
| **MPD>10mm** | 19 (13%) | 36 (23%) | 58 (30%) | 132 (37%) |
| **MPD 5-9** | 51 (35%) | 63 (40%) | 78 (40%) | 136 (38%) |
| **Cyst Size > 30 mm** | 63 (43%) | 85 (54%) | 114 (59%) | 187 (52%) |
| **Cyst wall Thickening** | 6 (4%) | 4 (2%) | 18 (9%) | 69 (19%) |
| **Lymphadenopathy** | 1 (1%) | 0 (0%) | 3 (2%) | 5 (1%) |
| **Elevated Ca19.9** | 25 (17%) | 30 (19%) | 16 (8%) | 13 (4%) |
| **Acute Pancreatitis** | 13 (9%) | 15 (10%) | 44 (23%) | 90 (25%) |
| **Diabetes Mellitus** | 0 (0%) | 3 (2%) | 7 (4%) | 22 (6%) |
| **No** **Risk Features** | 15 (10%) | 9 (6%) | 4 (2%) | 6 (2%) |
| **Total** | 208 (100%) | 275 (100%) | 419 (100%) | 842 (100%) |
| Abbreviations: Mural Nodule (MN); main pancreatic duct (MPD) | | | | |

**Figure S2: Distribution of IPMN subtypes (branch-duct (BD), main-duct (MD), and mixed-type (MT) IPMN) across three patient cohorts: those managed with surveillance (FU, follow-up), those undergoing upfront surgical resection (UF), and those undergoing surgery after more than 12 months of surveillance due to progression (PR, post-surveillance resection).**

**Figure S3: Distribution of pathological outcomes following IPMN resections over four time periods: <2006, 2006–2012, 2012–2017, and >2017**

**Table S5: Distribution of pathological outcomes (low-grade dysplasia, high-grade dysplasia, invasive carcinoma) in resected IPMN patients across the UF and PR cohorts, stratified by guideline period. This table corresponds to the data illustrated in Figure 2B.**

| **Variable** | **<2006** | | **2006-2012** | | **2012-2017** | | **>2017** | |
| --- | --- | --- | --- | --- | --- | --- | --- | --- |
|  | **UF** | **PR** | **UF** | **PR** | **UF** | **PR** | **UF** | **PR** |
| **other** | 0 (0%) | 0 (0%) | 1 (1%) | 0 (0%) | 9 (7%) | 4 (7%) | 14 (9%) | 22 (11%) |
| **LGD IPMN** | 66 (49%) | 5 (46%) | 60 (47%) | 14 (48%) | 43 (32%) | 22 (37%) | 39 (26%) | 35 (17%) |
| **HGD IPMN** | 24 (18%) | 2 (18%) | 24 (19%) | 9 (31%) | 44 (33%) | 12 (20%) | 28 (18%) | 56 (28%) |
| **IC IPMN** | 44 (33%) | 4 (36%) | 43 (34%) | 6 (21%) | 38 (28%) | 22 (37%) | 71 (47%) | 91 (45%) |
| ***Total*** | 134 (100%) | 11 (100%) | 128 (100%) | 29 (100%) | 134 (100%) | 60 (100%) | 152 (100%) | 204 (100%) |
